# A20 undermines alternative NF-κB activity and expression of anti-apoptotic genes in *Helicobacter pylori* infection

**DOI:** 10.1007/s00018-022-04139-y

**Published:** 2022-01-28

**Authors:** Michelle C. C. Lim, Gunter Maubach, Anna M. Birkl-Toeglhofer, Johannes Haybaeck, Michael Vieth, Mchael Naumann

**Affiliations:** 1 Institute of Experimental Internal Medicine, Otto Von Guericke University, Magdeburg, Germany; 2 Institute of Pathology, Neuropathology and Molecular Pathology, Medical University of Innsbruck, Innsbruck, Austria; 3 Institute of Pathology, Friedrich-Alexander University Erlangen-Nuremberg, Klinikum Bayreuth, Bayreuth, Germany

**Keywords:** ADP-heptose, TIFA, LTα_1_β_2_, NIK, p100, RelB

## Abstract

A hallmark of infection by the pathogen *Helicobacter pylori*, which colonizes the human gastric epithelium, is the simultaneous activation of the classical and alternative nuclear factor kappa-light-chain-enhancer of activated B cells (NF-κB) pathways, underlying inflammation and cell survival. Here, we report that the classical NF-κB target gene product A20 contributes to the negative regulation of alternative NF-κB signaling in gastric epithelial cells infected by *H. pylori*. Mechanistically, the de novo synthesized A20 protein interacts with tumor necrosis factor receptor-associated factor-interacting protein with forkhead-associated domain (TIFA) and thereby interferes with the association of TIFA with the NIK regulatory complex. We also show that alternative NF-κB activity contributes to the up-regulation of anti-apoptotic genes, such as baculoviral IAP repeat containing 2 (*BIRC2)*, *BIRC3* and B-cell lymphoma 2-related protein A1 (*BCL2A1)* in gastric epithelial cells. Furthermore, the observed over-expression of RelB in human gastric biopsies with type B gastritis and RelB-dependent suppression of apoptotic cell death emphasize an important role of the alternative NF-κB pathway in *H. pylori* infection.

**Supplementary Information:**

The online version contains supplementary material available at 10.1007/s00018-022-04139-y.

## Background

The human pathogen *Helicobacter pylori* is a Gram-negative bacterium which colonizes the gastric mucosal epithelium. Infection with *H. pylori* is common with a worldwide prevalence of 44% [1]. Unless treated, infection by *H. pylori* is persistent leading to chronic active gastritis. In addition, infection with *H. pylori* constitutes a major risk factor for the development of gastric neoplasia [2]. Importantly, the activation of NF-κB by *H. pylori* infection is the key contribution to the pro-inflammatory response and cell survival in the gastric mucosa [3].

In general, two branches of NF-κB activating pathways have been described: the classical and the alternative. The classical NF-κB pathway can be activated by a variety of stimuli, such as the pro-inflammatory cytokines interleukin-1β or tumor necrosis factor (TNF) and microbial pathogen-derived molecules [4]. The primary mechanism for classical NF-κB activation is the inducible IκB kinase complex (IKK)-mediated phosphorylation and thereafter degradation of IκBα. Consequently, the released classical NF-κB heterodimers, predominantly RelA/p50, translocate to the nucleus and regulate the expression of target genes including anti-apoptotic genes [5]. Notably, microbial pathogens have developed strategies to circumvent the NF-κB activity, underscoring the importance of the NF-κB pathway as a defense mechanism [6]. Activation of the alternative NF-κB pathway occurs upon stimulation of a subset of receptors belonging to the TNF receptor superfamily by their cognate ligands. These receptors include lymphotoxin β receptor (LTβR), fibroblast growth factor-inducible 14 (Fn14), receptor activator of NF-κB (RANK), cluster of differentiation 40 (CD40) and B-cell-activating factor receptor (BAFF-R) [7]. The NF-κB-inducing kinase (NIK) is the key signaling component that activates the alternative NF-κB pathway. In the cytosol of resting cells, the level of NIK is barely detectable due to its interaction with the NIK regulatory complex consisting of TRAF3, TRAF2 and cIAP1 or cIAP2 (cIAP1/2) [8]. Specifically, the interaction of TRAF3 with TRAF2 enables the TRAF2-bound cIAP1/2 to catalyze the ligation of K48-linked polyubiquitin to TRAF3-bound NIK, ensuring the constant degradation of NIK by the 26S proteasome. Activation of an appropriate receptor leads to the recruitment of the NIK regulatory complex to the cytoplasmic region of the receptor. Depending on the stimulus, separate mechanisms are involved that lead to the disruption of the NIK regulatory complex; as a result, NIK is released and accumulates in the cytosol [7, 9, 10]. Stabilized NIK activates IKKα by phosphorylation and both proteins bind to the RelB/p100 dimer [11]. Next, IKKα phosphorylates p100, leading to its proteolytic processing to p52 and the subsequent nuclear translocation of the RelB/p52 heterodimer [7]. In addition, classical NF-κB regulates the transcription of RelB [12] and p100 [13], and promotes the generation of more RelB/p52 dimers.

*H. pylori* induces classical NF-κB in human gastric epithelial cells in a type IV secretion system (T4SS)-dependent and cytotoxin-associated gene A (CagA)-independent manner [14, 15]. Recently, the ADP-L-glycero-β-d-manno-heptose (ADP-heptose) of Gram-negative bacteria, a metabolic precursor of lipopolysaccharide, has been identified as the predominant trigger of the classical NF-κB pathway via binding to the cytosolic alpha-protein kinase 1 (ALPK1) [16]. The catalytic activity of ALPK1 is required for the self-oligomerization of tumor necrosis factor receptor-associated factor (TRAF)-interacting protein with forkhead-associated domain (TIFA) [17], which together with TRAF6 and transforming growth factor β-activated kinase 1 (TAK1) activates the classical NF-κB pathway [18]. The ADP-heptose – ALPK1 – TIFA signaling module is also essential for alternative NF-κB activation by *H. pylori*. We have recently shown that the binding of TIFA to the NIK regulatory complex facilitates the proteasome-dependent transient turnover of cIAP1, resulting in the stabilization of NIK [18].

An important target gene of the classical NF-κB is *TNFAIP3* which encodes A20. The A20 protein contains an N-terminal ovarian tumor domain, which has a deubiquitinylase activity, and C-terminal zinc finger (ZnF) domains, of which the ZnF4 and ZnF7 domains function as ubiquitin-binding domains [19]. Herein, A20 down-regulates classical NF-κB signaling either in a catalytic or a non-catalytic manner [20–24].

First attempts to infect the gastric epithelium under conditions close to the in vivo situation have been made using 3D organoids and 2D organoid monolayers. These data showed that *H. pylori* infection leads to a reliable induction of NF-κB signaling and expression of its target genes, comparable to that in gastric cancer cell lines [25–27]. Unlike certain stimuli that induce prominently either the classical or alternative NF-κB pathway, our studies showed that infection by *H. pylori* induces robustly both NF-κB pathways [28, 29]. Thus, we took advantage of this phenomenon in *H. pylori*-infected cells to study the function of classical NF-κB-induced A20 in the alternative NF-κB pathway. Taken together, our study establishes that classical NF-κB-dependent up-regulation of A20 suppresses not only classical but also alternative NF-κB, and blocks expression of specific anti-apoptotic genes.

## Results

### A20-deficient gastric epithelial cells show enhanced alternative NF-κB activation in ***H. pylori*** infection

**Fig. 1:**
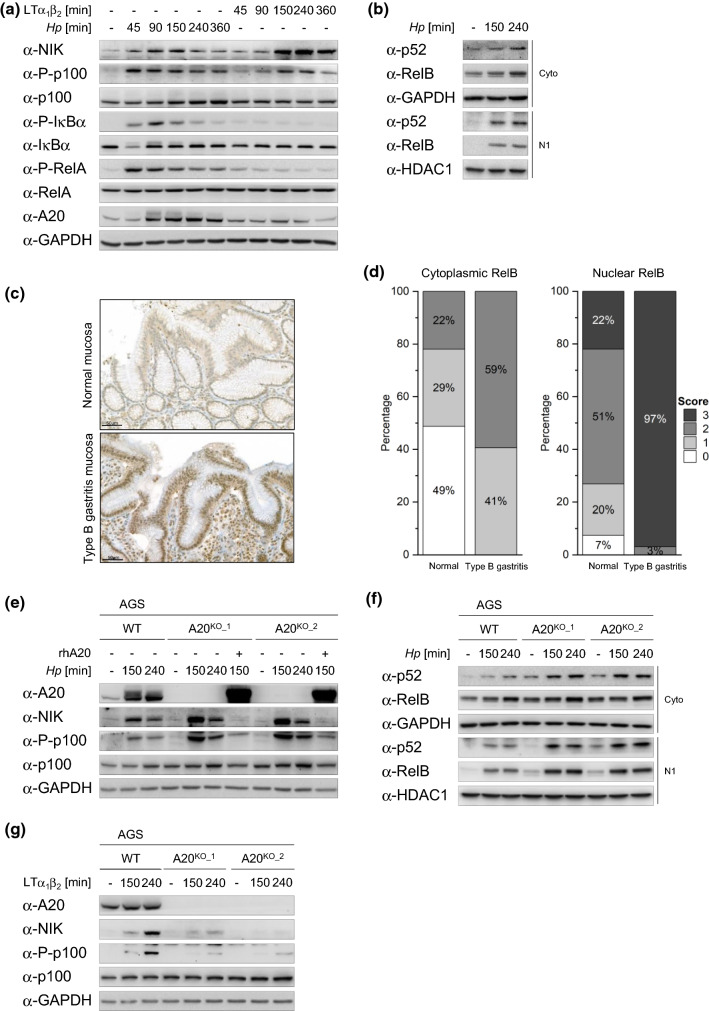
A20 participates in the negative regulation of alternative NF-κB activation in *H. pylori* infection. **a** AGS cells were infected with *H. pylori* or stimulated with 30 ng/ml LTα_1_β_2_. Total cell lysates were analyzed for the indicated proteins by immunoblot (IB). **b** AGS cells were infected with *H. pylori*. The cytosolic (Cyto) and soluble nuclear (N1) fractions were analyzed by IB. **c** Representative immunohistochemical micrographs of RelB-stained normal (upper panel) or type B gastritis (lower panel) gastric mucosal specimens. See scale bar for magnification. **d** Left graph shows moderate cytoplasmic RelB staining intensity in type B gastritis cases, while weaker staining intensity was observed in healthy control gastric specimens, *p* < 0.001. Right graph shows high nuclear RelB staining intensity in the case of type B gastritis (*n* = 32) compared to normal gastric mucosa (*n* = 41), *p* < 0.001. **e** Wild-type AGS (WT) and two clones of A20-deficient AGS cells (A20^KO_1^ and A20^KO_2^) were infected with *H. pylori*. Total cell lysates were analyzed for the indicated proteins by IB. **f** WT, A20^KO_1^ and A20^KO_2^ AGS cells were infected with *H. pylori*. The cytosolic (Cyto) and soluble nuclear (N1) fractions were analyzed by IB. **g** WT, A20^KO_1^ and A20^KO_2^ AGS cells were treated with 30 ng/ml LTα_1_β_2_. Total cell lysates were analyzed for the indicated proteins by IB. A representative blot of at least two experiments was shown

In *H. pylori*-infected AGS cells, we observed an early (within 45 min) inducible phosphorylation of IκBα and RelA, followed by a significant increase in de novo synthesized A20, indicating classical NF-κB activation (Fig. 1a). Concomitantly, we also observed the activation of alternative NF-κB as shown by the transient accumulation of NIK (peaked at 2.5 h) and the inducible phosphorylation of p100 (Fig. 1a), as well as the nuclear translocation of p52 and RelB, which are the essential dimer-forming subunits of alternative NF-κB (Fig. 1b). In comparison, stimulation by LTα_1_β_2_ led to a slower and longer kinetic of alternative NF-κB activation (Fig. 1a). Contrary to infection by *H. pylori*, LTα_1_β_2_ treatment triggered negligible classical NF-κB signaling, therefore no significant up-regulation in the expression of A20 was observed (Fig. 1a). *H. pylori*-induced alternative NF-κB activation was also observed in the epithelial cell lines NCI-N87 and HKC-8 (Suppl. Fig. S1a, b). To corroborate our in vitro data, we studied RelB expression in human gastric tissue biopsy samples with no pathological changes and samples from patients with *H. pylori*-associated type B gastritis by immunohistochemistry. RelB was evident in both the nuclei and the cytoplasm of epithelial cells in the gastric mucosa (Fig. 1c). RelB staining was mainly observed as nuclear staining in epithelial cells and the tissue intensity score was significantly higher in type B gastritis than in non-*H. pylori*-infected tissue (Fig. 1d, right graph), indicating enhanced alternative NF-κB signaling that may be pathologically relevant. Moderate cytoplasmic RelB staining was also seen in type B gastritis cases, whereas weak staining intensities were detected in samples with no pathological changes (Fig. 1d, left graph). These observations could be attributed to the up-regulation of RelB expression by activation of the classical NF-κB [12].

To explore the role of A20 in the alternative NF-κB activation induced by *H. pylori*, we used A20-deficient AGS cells generated by CRISPR/Cas9, which are henceforth referred to as A20^KO^ cells. Control AGS cells are referred to as wild-type cells. In A20^KO^ cells infected with *H. pylori* strain P1, we observed augmented alternative NF-κB activation as reflected by the significantly increased accumulation of NIK, phosphorylation of p100 as well as nuclear translocation of p52 and RelB (Fig. 1e, f). This observed difference in the activation of alternative NF-κB in wild-type and A20^KO^ cells was corroborated by infection with another *H. pylori* strain P12 (Suppl. Fig. S2a). Importantly, A20^KO^ cells complemented by transfection of recombinant human A20 protein showed almost a complete abrogation of alternative NF-κB activation (Fig. 1e). Similarly, AGS and NCI-N87 cells depleted of A20 by siRNA transfection showed also markedly stronger alternative NF-κB activation (Suppl. Fig. S2b, c). On the contrary, LTα_1_β_2_-induced alternative NF-κB activation was significantly reduced in A20^KO^ cells compared to wild-type cells (Fig. 1g), an observation that is in agreement with the report by Yamaguchi and colleagues [30]. Taken together, our results indicate that in *H. pylori* infection, A20 contributes to the abatement of alternative NF-κB signaling, resulting in less RelB/p52 in the nucleus.

### A20 interacts with NIK regulatory complex-bound TIFA in *H. pylori* infection

**Fig. 2:**
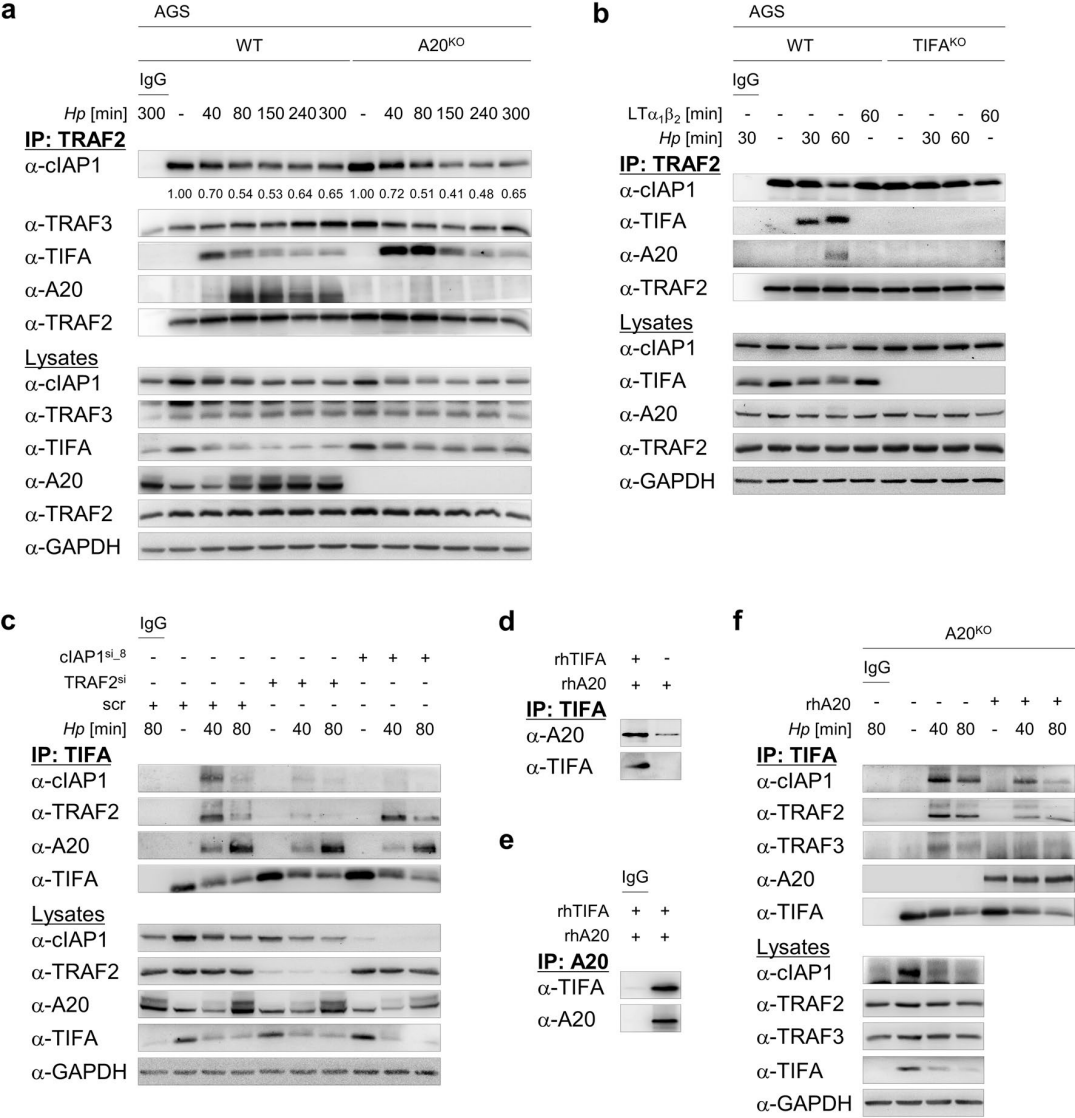
Inhibition of *H. pylori*-mediated alternative NF-κB signaling pathway by A20 requires TIFA. **a** WT and A20^KO_1^ AGS cells were infected with *H. pylori*. Total cell lysates were harvested and IP was performed using an antibody against TRAF2 or isotype-matched IgG (IgG). The numbers indicate the band intensities of cIAP1 (normalized to the respective band intensities of TRAF2) of *H. pylori*-infected samples relative to uninfected control. **b** WT and TIFA-knockout (TIFA^KO^) AGS cells were infected with *H. pylori* or treated with 30 ng/ml LTα_1_β_2_. Total cell lysates were subjected to IP as in (**a**). **c** AGS cells were transfected with non-target-specific siRNA (scr) or siRNAs targeting TRAF2 (TRAF2^si^) or cIAP1 (cIAP1^si_8^) for 48 h prior to infection with *H. pylori*. Total cell lysates were used for IP with an antibody against TIFA or isotype-matched IgG. **d** Following incubation of recombinant human A20 and TIFA proteins in vitro, IP was performed using an antibody against TIFA. **e** as in (**d**) but IP was performed using an antibody against A20 or isotype-matched IgG. **f** A20^KO_1^ AGS cells were infected with *H. pylori* and IP was performed as in (**c**). IB analyses were performed with TIFA-immunoprecipitates without and with one hour incubation with 100 ng recombinant A20 protein prior to elution of immunoprecipitates. A representative blot of at least two experiments was shown

We have recently shown that TIFA is crucial for *H. pylori*-induced alternative NF-κB activation. Specifically, the binding of TIFA to the NIK regulatory complex (TRAF3/TRAF2/cIAP1) supports the proteasome-dependent transient turnover of cIAP1 and the ensuing accumulation of NIK [18]. Thus, we asked whether A20 functions at this juncture in the alternative NF-κB signaling pathway. Intriguingly, in a TRAF2 IP, we found at early time points after *H. pylori* infection a stronger and longer lasting association of TIFA with TRAF2 in A20^KO^ cells, which correlated with decreasing amounts of cIAP1 (Fig. 2a). Further, an inducible interaction of TRAF2 and A20 was triggered upon *H. pylori* infection (Fig. 2a, b) but not in response to LTα_1_β_2_ treatment (Fig. 2b). In TIFA-knockout cells, no A20 was co-immunoprecipitated with TRAF2 (Fig. 2b). These observations are consistent with our previous findings that the association of TIFA with the NIK regulatory complex is specific for *H. pylori* infection [18] and also suggest that the interaction of A20 with the NIK regulatory complex depends on TIFA.

To narrow down the direct interaction partner of A20 in the NIK regulatory complex, we performed siRNA-mediated depletion of TRAF2, cIAP1 or TRAF3 followed by a TIFA IP. Strikingly, knockdown of TRAF2, cIAP1 or TRAF3 did not prevent the binding of A20 to TIFA in response to *H. pylori* infection (Fig. 2c, Suppl. Fig. S3a). When we incubated recombinant human A20 and TIFA proteins together in vitro, we could immunoprecipitate the A20 protein in a TIFA IP (Fig. 2d) and vice versa (Fig. 2e). These results led us to speculate that this interaction is of relevance to the function of A20 in the suppression of TIFA-dependent alternative NF-κB activity. To test this hypothesis, we infected A20^KO^ cells and performed a TIFA IP. Immunoblot analyses of the TIFA-immunoprecipitates revealed the association of components of the NIK regulatory complex with TIFA (Fig. 2f, lanes 2–4). However, when we incubated the IP samples (beads with TIFA-immunoprecipitates) with recombinant human A20 protein for one hour prior to immunoblot analyses of the TIFA-immunoprecipitates, we observed a reduction in the abundance of TIFA-associated cIAP1, TRAF2 and TRAF3 (Fig. 2f, lanes 5–7). Thus, our results demonstrate that upon *H. pylori* infection, A20 binds to TIFA within the NIK regulatory complex that in turn leads to the displacement of TIFA.

### Alternative NF-κB signaling promotes the expression of anti-apoptotic genes during *H. pylori* infection

**Fig. 3:**
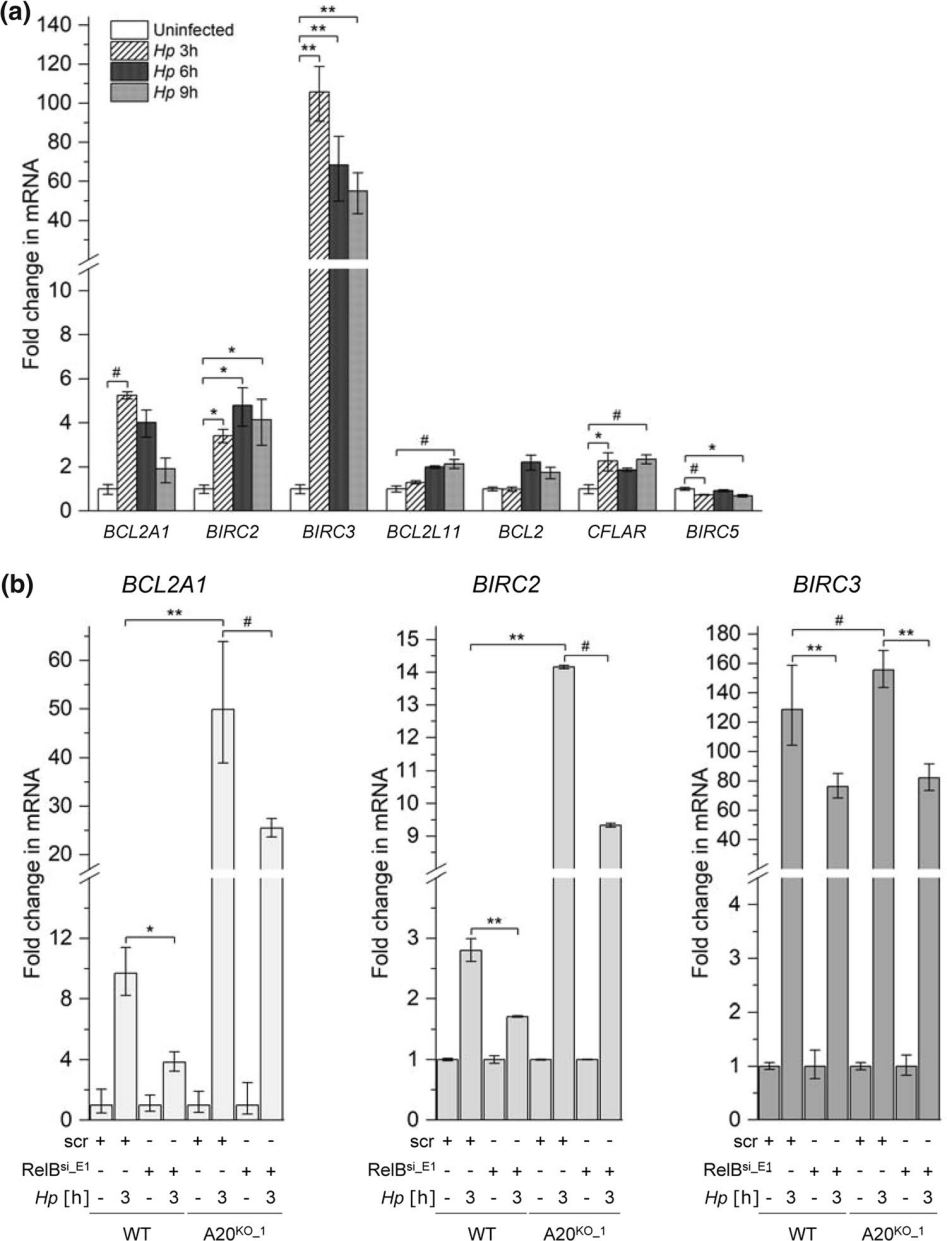
A20 inhibits specific alternative NF-κB-regulated anti-apoptotic genes in *H. pylori* infection. **a** Total RNA was isolated after *H. pylori* infection of AGS cells. Changes in the expression of the indicated transcripts were analyzed by quantitative PCR. **b** WT and A20^KO_1^ AGS cells were transfected with non-target-specific siRNA (scr) or siRNA targeting RelB (RelB^si_E1^) for 48 h prior to infection by *H. pylori* for 3 h. Total RNA was isolated and analyzed as in (**a**). **a** and **b** Data are normalized to the *RPL13A* housekeeping gene and expressed as fold change in mRNA expression relative to uninfected cells. Triplicate determinations of each experiment was performed. Error bars denote means ± SD of at least two independent experiments. ^#^*p* < 0.05, *p < 0.01 and **p < 0.001

Classical and alternative NF-κB signaling regulates distinct as well as overlapping target genes involved in many cellular processes including cell survival [31]. To analyze the impact of *H. pylori*-induced alternative NF-κB on cell survival, we first carried out quantitative PCR to analyze the transcription of anti-apoptotic genes, *BCL2L11* (BIM), *BCL2* (BCL2), *BIRC2* (cIAP1), *BCL2A1* (BCL2A1), *CFLAR* (cFLIP), *BIRC3* (cIAP2) and *BIRC5* (survivin), known to be regulated by NF-κB. Infection by *H. pylori* led to a striking albeit transient up-regulation of *BIRC2*, *BIRC3* and *BCL2A1* (Fig. 3a). In response to *H. pylori* infection, RelB-depleted cells showed a lower expression of these three genes, suggesting that they are regulated at least in part by alternative NF-κB (Fig. 3b). Further, we examined whether the inhibitory effect of A20 on the alternative NF-κB pathway could have an impact on the expression of these three anti-apoptotic genes. Upon *H. pylori* infection, the up-regulation of *BIRC2*, *BIRC3* and *BCL2A1* was further increased in A20^KO^ cells compared to wild-type cells (Fig. 3b). This is most likely due to the missing inhibitory effect of A20 on not only the alternative but also the classical NF-κB signaling pathway. Importantly, RelB-depleted A20^KO^ cells (compromised in alternative NF-κB) showed attenuated up-regulation of these anti-apoptotic genes compared to A20^KO^ cells (Fig. 3b). This diminished up-regulation represents the contribution of alternative NF-κB to the expression of these anti-apoptotic genes.

**Fig. 4:**
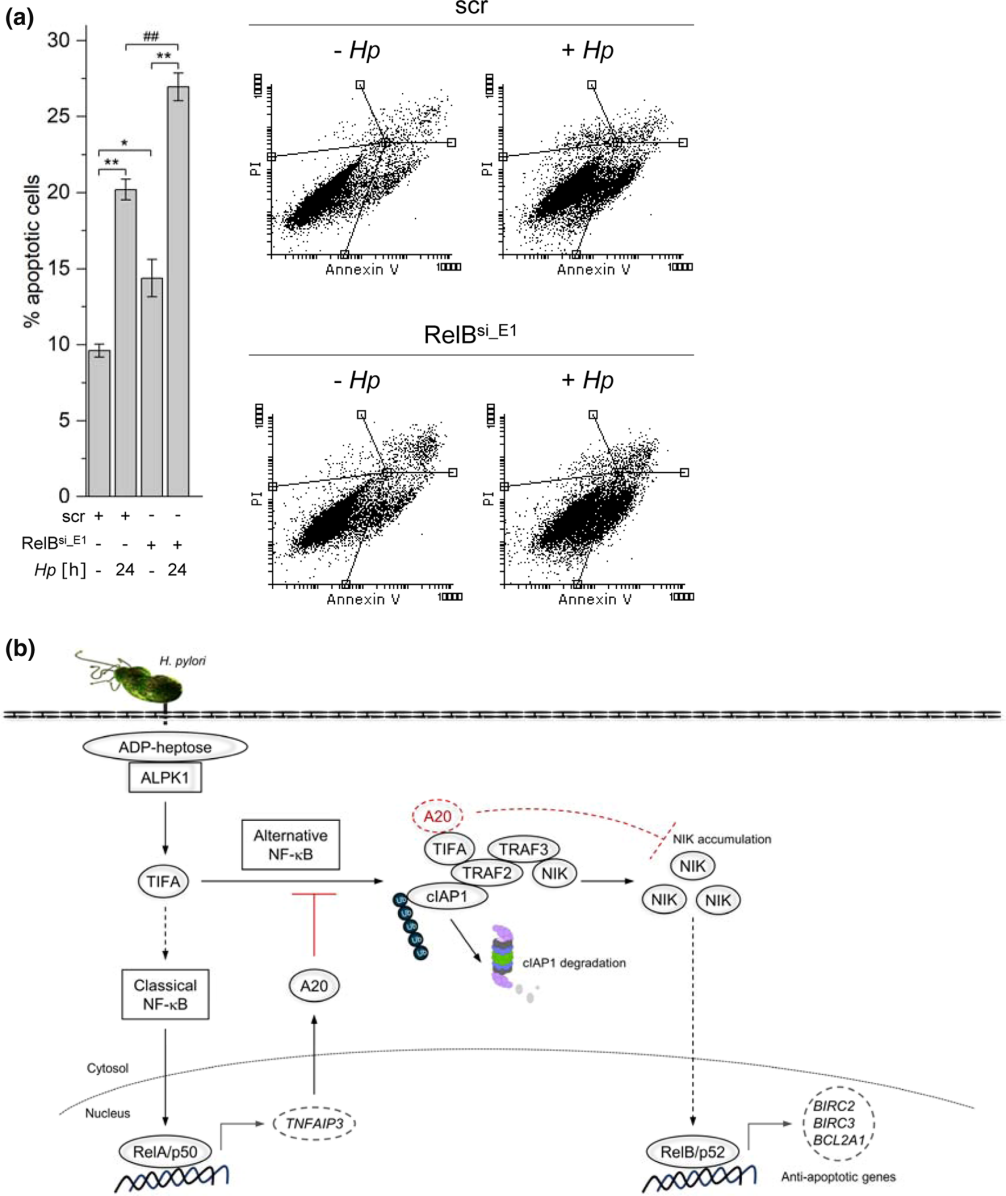
Alternative NF-κB signaling contributes to cell survival in *H. pylori* infection. **a** AGS cells were transfected with non-target-specific siRNA (scr) or siRNA targeting RelB (RelB^si_E1^) for 48 h. After *H. pylori* infection for 24 h, cells were harvested and stained with annexin V-FITC/PI, and analyzed by flow cytometry. A graph showing data from three independent experiments is depicted (means ± SD). ^##^*p* < 0.005, *p < 0.01 and **p < 0.001. Representative dot plots are shown for each treatment. **b** A proposed model for A20’s role in the negative regulation of alternative NF-κB signaling pathway in *H. pylori* infection. *H. pylori*-induced activation of the classical NF-κB pathway results in the up-regulation of A20 expression. A20 binds to TIFA in the TIFA/NIK regulatory complex, which in turn destabilizes the association of TIFA with the complex. This restores the stability of cIAP1 in the complex and this TIFA-free NIK regulatory complex recovers the ability to mediate the proteasomal degradation of NIK, leading to the shutdown of alternative NF-κB signaling

We proceeded to determine apoptotic cell death by flow cytometric analysis of cells stained with annexin V-FITC and propidium iodide (PI). After *H. pylori* infection, we observed an increase in the percentage of apoptotic cells in control and RelB-depleted cells compared to their uninfected counterparts, 9.6 to 20.2% and 14.4 to 27.0%, respectively (Fig. 4a), indicating that cells with reduced expression of RelB were even more sensitive to *H. pylori*-induced apoptosis. Taken together, our data imply that A20 undermines cell survival to a certain extent because of its inhibitory effect on alternative and classical NF-κB-dependent regulation of anti-apoptotic genes.

## Discussion

Infection by *H. pylori*, which mediates in parallel prominent activation of the classical as well as the alternative NF-κB signaling pathways, provides us with a unique opportunity to examine the impact of A20, a classical NF-κB-induced gene product, on the regulation of the alternative NF-κB pathway. In this study, we demonstrate that A20 participates in the negative regulation of alternative NF-κB signaling and gene regulation during *H. pylori* infection (Fig. 4b). Accordingly, A20 interacts with TIFA within the TIFA/NIK regulatory complex, which in turn destabilizes the interaction of TIFA with the complex. This restores the stability of cIAP1 in the complex, an observation that is consistent with our previous findings [18]. This TIFA-free NIK regulatory complex recovers the ability to mediate the proteasomal degradation of NIK, leading to the shutdown of alternative NF-κB signaling.

In a previous publication [29], we have established by knockdown experiments that the LTβR is involved in *H. pylori*-induced alternative NF-κB. However, the role of the LTβR in contributing to the activation of the alternative NF-κB is not clear. Here, an internalization of the LTβR as described by Ganeff et al. [32] could be a way where the receptor is involved independent of its ligand. The cytoplasmic binding of TRAF2/3 to the internalized LTβR might contribute to the liberation of NIK from the NIK regulatory complex [9] after cIAP1 is unable to further ubiquitinylate NIK [18]. Further, an earlier study reported that A20 was involved, independent of its catalytic activities, in the activation of LTβR, Fn14, and RANK-mediated alternative NF-κB signaling in mouse embryonic fibroblasts and MDA-MB-231 cells [30]. These authors reported that in ligand–receptor-mediated activation of alternative NF-κB, the binding of A20 to cIAP1 results in the disruption of the interaction between TRAF2 and TRAF3. This in principle leads to the disintegration of the NIK regulatory complex so that NIK could accumulate and activate alternative NF-κB signaling. In the course of this study and our previous observations [18], we showed that involvement of the ADP-heptose – ALPK1 – TIFA signaling module is the feature that distinguishes the activation of alternative NF-κB in *H. pylori* infection from ligand–receptor-mediated signaling. Consistently, we show in the current study that A20 requires TIFA to fulfill its inhibitory function on *H. pylori*-induced alternative NF-κB signaling. Therefore, the *H. pylori*-dependent binding of TIFA to the NIK regulatory complex might mask the binding site of A20 on cIAP1, which in turn drives the response of NF-κB-induced A20 toward a different binding partner, and this also explains the distinct functions of A20 reported here and by Yamaguchi et al*.* [30].

Most knowledge about the alternative NF-κB pathway has been garnered in connection with lymphoid organogenesis and functions of immune cells, such as B cells and dendritic cells [33]. However, receptors which trigger alternative NF-κB activation including LTβR, Fn14 and RANK are also expressed in non-immune cells, such as fibroblasts, endothelial and epithelial cells [34–36]. Whether the alternative NF-κB signaling in non-immune cells has a function in cell survival has not been investigated in detail. We showed that alternative NF-κB activity contributes to the up-regulation of survival genes *BIRC2*, *BIRC3* and *BCL2A1* in epithelial cells. Furthermore, we found a positive correlation between the absence of RelB and the increase in apoptotic epithelial cells upon *H. pylori* infection, suggesting that alternative NF-κB activity is pro-survival. This represents in the pathophysiology of *H. pylori* infection an obvious advantage for the colonizing pathogen to prevent excessive disruption of the integrity of the epithelial barrier. Supporting this notion is an earlier study showing that LTβR-driven alternative NF-κB signaling has a protective role in intestinal epithelial cells by inducing the production of IL-23 to promote cell survival [37]. Our study, therefore, expands the understanding of the impact of alternative NF-κB activity on cell survival during the colonization of gastric epithelial cells by *H. pylori*. Further investigation is warranted to address comprehensively the cross-talk between this pro-survival function of *H. pylori*-induced alternative NF-κB and its other function in facilitating the recruitment of immune cells during infection [38].

On the one hand, we have previously reported that A20 interacts with caspase-8 and suppresses the processing of caspase-8, thereby counteracting *H. pylori*-associated caspase-8-dependent apoptotic cell death [39]. On the other hand, we showed in this study that A20 suppresses classical and alternative NF-κB-regulated anti-apoptotic genes, and thereby promotes *H. pylori*-associated apoptotic cell death. The apoptosis response in host cells is a double-edged sword for *H. pylori* because while the bacterium benefits from the nutrients that are provided by the apoptotic host cells, disintegration of the host cells also diminishes its colonization niche. Future studies that focus on elucidating how these opposing functions of A20 are regulated (spatially, temporally and possible involvement of other participating molecules) could reveal ways to better appraise the benefits for *H. pylori* and broaden our understanding on how these might contribute to cellular transformation and gastric cancer.

## Materials and methods

### Cell culture conditions

AGS (ATCC® CRL-1739™), NCI-N87 (ATCC® CRL-5822™), HKC-8 (RRID:CVCL_Y910), A20- and TIFA-knockout [18] cells were routinely cultivated in RPMI-1640 medium (Gibco™, 21875–034) supplemented with 10% fetal calf serum (FCS) at 37 °C in a 5% CO_2_ humidified incubator. Cells were seeded at a density of 6 × 10^5^ per 60 mm culture dish or 2.7 × 10^6^ per 100 mm culture dish for infection with *H. pylori* or treatment with recombinant human LTα_1_β_2_ (R&D Systems, 8884-LY). Cell culture medium was changed to fresh RPMI-1640 medium supplemented with 0.2% FCS 16–20 h prior to infection or treatment of the cells.

### Bacterial culture

The *H. pylori* wild-type P1 or P12 (used for Suppl. Fig. S2a only) strain was streaked from − 80 °C stock onto agar-plates containing 10% horse serum and 10 µg/ml vancomycin, and cultivated under microaerophilic conditions for 48–72 h. Bacteria was re-plated and cultivated for 48 h before use in experiments. The bacterial suspension used for infection of cells was prepared in phosphate-buffered saline containing Mg^2+^ and Ca^2+^ (PBS). The optical density at 550 nm was measured to determine the number of bacteria in the suspension. Infection of bacteria was performed at a multiplicity of infection (MOI) 100.

### Generation of A20-knockout AGS cell lines using CRISPR/Cas9

The knockout of A20 in AGS cells was performed using reagents from IDT as described in [18]. The Alt-R® CRISPR-Cas9 crRNA Hs.Cas9.TNFAIP3.1.AB 5’-AACCATGCACCGATACACAC TGG -3’ (IDT) was used. The control AGS cells used have undergone the same CRISPR/Cas9 procedure but were not successful for the depletion of A20.

### Transfection of siRNAs and recombinant proteins

Transfection of siRNAs was performed using the METAFECTENE® SI^+^ kit (Biontex Laboratories, T100-1.0) according to the manufacturer’s protocol. Briefly, 24 h prior to siRNA transfections, cells were seeded at a density of 1.5 × 10^5^ per 60 mm culture dish or 0.5 × 10^6^ per 100 mm culture dish. Cell culture medium was changed to fresh RPMI-1640 supplemented with 10% FCS prior to transfection. The siRNAs were used at a final concentration of 40 nM for A20, 30 nM for TRAF3, TRAF2 and cIAP1, and 20 nM for RelB. As a control for non-specific effects of the transfection procedure, equimolar of scrambled (non-target-specific) siRNA was used for the respective experiments. The siRNAs were transfected into the cells for 24 h before the cell culture medium was changed to fresh RPMI-1640 medium supplemented with 0.2% FCS or 10% FCS (for experiments in Fig. 4a) 16–20 h prior to *H. pylori* infection. The following siRNAs were used: scrambled siRNA (Qiagen, SI03650318); A20^si_5^ (Dharmacon, J-009919–05-0005); A20^si_9^ (Qiagen, SI05018601); RelB^si_E1^ (Eurofins, 5’-GACUGCACCGACGGCAUCU-dTT-3’ [40]); TRAF3 (Santa Cruz Biotechnology, sc-29510); TRAF2 (Santa Cruz Biotechnology, sc-29509); and cIAP1 (Qiagen, SI02654442).

Transfection of recombinant human A20 protein (BPS Bioscience, 80408) was performed using reagents from the Lipofectamine® CRISPRMAX transfection reagent kit (Invitrogen, CMAX00015). Twenty-four hours prior to protein transfection, cells were seeded at a density of 6 × 10^5^ per 60 mm culture dish. The cell culture medium was changed to fresh RPMI-1640 medium supplemented with 0.2% FCS prior to transfection. One microgram recombinant protein was combined with 5 µl Cas9 PLUS™ reagent in 500 µl Opti-MEM (Gibco™, 31985070) and incubated at room temperature for 5 min. Six microliters of CRISPRMAX transfection reagent diluted in 500 µl Opti-MEM was combined with the diluted Cas9 PLUS™ reagent /recombinant protein solution and incubated at room temperature for 20 min before adding drop-wise to the cells in the dishes. After 16–20 h, the cells were infected with *H. pylori*.

### SDS-PAGE and immunoblotting

For preparation of total cell lysates, cells were washed twice in ice-cold PBS and 100 μl or 500 μl of lysis buffer (50 mM Tris/HCl pH 7.5, 150 mM NaCl, 5 mM EDTA, 10 mM K_2_HPO_4_, 10% glycerol, 1% Triton X-100 and 0.5% NP-40) containing phosphatase inhibitors (1 mM sodium vanadate, 1 mM sodium molybdate, 20 mM sodium fluoride, 10 mM sodium pyrophosphate, 1 mM AEBSF and 20 mM 2-phosphoglycerate) and protease inhibitor mix (Roche Diagnostics, 34044100) was added to a 60 mm or 100 mm dish, respectively. Cells were scraped, transferred into microtubes and incubated on ice for 15 min. For preparation of cell lysates for IP, the cell lysates were sheared through a 19G needle several times prior to incubation on ice. Cleared cell lysates were obtained after centrifugation at 13,000 rpm for 15 min at 4 °C. The subcellular fractionation of cells into cytosolic and soluble nuclear fractions was performed as described in previous work [41].

Protein concentration was determined using the BCA protein assay kit (ThermoFisher Scientific, 23225). SDS-PAGE was performed in Tris–Glycine gels and transferred onto PVDF membranes (Merck Millipore). The membranes were blocked for 1 h at room temperature using 5% skim milk in TBS containing 0.1% Tween (TBS-T). The membranes were incubated overnight with primary antibodies at the appropriate dilutions in either 5% BSA or 5% skim milk in TBS-T at 4 °C on a rocking platform. The membranes were washed thrice in TBS-T and incubated with the appropriate HRP-conjugated secondary antibody for 1 h at room temperature at a dilution of 1:6000 in 5% skim milk in TBS-T, followed by three washes in TBS-T. The membranes were developed using a chemiluminescent HRP substrate (Merck Millipore, WBKLS0500). The band pattern was visualized using the ChemoCam Imager (Intas).

Antibodies used in this work were as follows: α-A20 (5630, used for detection in IB after TRAF2 IP); α-cIAP1 (7065); α-IκBα (4812); α-NF-κB2 p100/p52 (4882); α-NIK (4994); α-TIFA (61,358); α-TRAF3 (61095); α-phospho-IκBα (9246); α-phospho-p100 (4810); and α-phospho-RelA (3031) from Cell Signaling Technology; α-A20 (sc-166692), α-HDAC1 (sc-7872), α-RelA (sc-81334), α-RelB (sc-226) and α-TRAF2 (sc-136999) from Santa Cruz Biotechnology; α-TRAF3 (Invitrogen, 700121); and α-GAPDH (Merck Millipore, MAB374). The secondary anti-rabbit-HRP (711–036-152), anti-mouse-HRP (715–036-151), anti-light chain-specific rabbit-HRP (211–032-171) and anti-light chain-specific mouse-HRP (115–035-174) antibodies were from Jackson ImmunoResearch Laboratories. The anti-light chain-specific secondary antibodies were used for analysis of immunoblots after IPs.

### Immunoprecipitation

Two to four milligrams of total cell lysate was incubated with 1 µg antibody against the targeted protein or isotype-matched IgG overnight. Protein A/G magnetic beads (ThermoFisher Scientific, 88803) were added followed by incubation for 1 h. Both incubation steps were carried out at 4 °C on a rotator at 7 rpm. The beads with immunoprecipitates were washed three times in lysis buffer and twice in PBS. Elution of immunoprecipitated proteins from the beads was achieved by incubation with 2 × Laemmli sample buffer for 20 min at room temperature. Eluate was transferred to a clean microtube and heated for 5 min at 95 °C prior to SDS-PAGE and immunoblotting.

For the experiments in Fig. 2, panels d and e, 50 ng recombinant human A20 and TIFA (Novus Biologicals, NBP1-99103-50ug) proteins, respectively, were combined in 500 μl PBS, and incubated for 1 h. IP was performed by incubation with 1 μl α-TIFA or 1 µg α-A20 (Santa Cruz Biotechnology) antibody for 1 h. Both incubation steps were carried out at 4 °C on a rotator at 7 rpm. Subsequent steps were the same as described in the previous paragraph.

For the experiments in Fig. 2, panel f, after the last washing step of the beads with immunoprecipitates, these were resuspended in 500 μl PBS and divided into two aliquots. Elution of immunoprecipitates was performed for one aliquot. One-hundred nanograms recombinant human A20 protein was added to the other aliquot and rotated at 7 rpm for 1 h at 4 °C. The beads with immunoprecipitates were washed two times in lysis buffer followed by elution of immunoprecipitates.

### Flow cytometry

Analysis of cells after annexin V/propidium iodide (PI) staining was performed using the CyFlow® Space flow cytometer (Sysmex Partec) and quantified using the Flowing Software (version 2.5.1). Cells were harvested using trypsin and stained using the Annexin-V Apoptosis Detection kit (MabTag, AnxF100PI) according to the manufacturer’s protocol. A gating for single cells was performed and 1 × 10^4^ cells were counted. For the quantification of cell death, annexin V-positive and double positive (annexin V- plus PI-stained) cells were added together to give the percentage of apoptotic cells.

### Quantitative PCR

Total RNA was isolated using the NucleoSpin® RNA Plus kit (Macherey–Nagel). The total RNA was reverse-transcribed into cDNA using the RT^2^ HT First Strand kit (Qiagen). Quantitative PCR was performed using the primer sets targeted against *BCL2L11*, *BCL2*, *BIRC2*, *BCL2A1*, *CFLAR*, *BIRC3*, *BIRC5* and *RPL13A* (endogenous control gene for normalization) provided in the Human Apoptosis Primer Library masterplate (RealTimePrimers, HPA-1). The Comparative C_T_ Method (ΔΔC_T_) was used to quantify relative changes of the target mRNA.

### Immunohistochemistry

Immunohistochemical staining for RelB (1:100 dilution) was performed on paraffin-embedded human gastric tissue sections (3 μm) of biopsies (Ethics approval: 347_20 Bc) from *H. pylori*-negative patients with normal gastric mucosa and *H. pylori*-positive patients with chronic inflammation using the VENTANA BenchMark XT system and the *ultra*View Universal DAB detection kit according to the manufacturer’s instructions (Ventana Medical Systems). For epitope retrieval, the Benchmark ULTRA CC1 solution (‘Mild’ heat-induced epitope retrieval standard pre-programmed protocol) was used. Image acquisition was performed with Hamamatsu Nanozoomer S360 scanner and software (Hamamatsu). Staining was evaluated independently by two board-certified pathologists (JH and MV) and the staining intensity was scored as follows: score 0 was assigned to no staining, score1 to weak, score 2 to moderate, and score 3 to strong staining according to the Remmele IR score [42].

### Densitometric analysis

The densitometric quantification of cIAP1 band intensities in Fig. 2a was performed using the ImageJ software according to Schneider et al. [43]. For normalization purpose, the values of the cIAP1 band intensities were divided by the values of the respective TRAF2 band intensities. The numbers indicate the normalized band intensities of cIAP1 in *H. pylori*-infected samples relative to uninfected control.

### Statistics

All quantitative data were analyzed using OriginPro 2020b and are presented as means ± SD. Time-dependent changes during quantitative PCR studies (Fig. 3a) were tested for significance using one-way ANOVA and Bonferroni’s post hoc test. All other quantitative data were tested for significance using two-sample Student’s *t* test. *p* < 0.05 was regarded as statistical significant. For immunohistochemical analysis, the Mann–Whitney *U* test was performed (GraphPad 9.0.0). *p* < 0.001 was regarded as statistical significant.

## Supplementary Information

Below is the link to the electronic supplementary material.

Supplementary file1 (PDF 2769 KB)

## Data Availability

All data generated and analyzed during the current study are included in this published article and its additional files.
