# Supplementary material for: A20 undermines alternative NF-κB activity and expression of anti-apoptotic genes in Helicobacter pylori infection
Source: Cell Mol Life Sci. 2022 Jan 28;79(2):102. doi: 10.1007/s00018-022-04139-y (PMC8799570; doi:10.1007/s00018-022-04139-y)

A20 undermines alternative NF-κB activity and expression of anti-apoptotic genes in *Helicobacter pylori* infection

Michelle C.C. Lim[1], Gunter Maubach[1], Anna M. Birkl-Toeglhofer[2], Johannes Haybäck[2], Michael Vieth[3], Michael Naumann[1#]

Supplementary Figure 1: Infection by *H. pylori* activates the alternative NF-κB pathway. HKC-8 and NCI-N87 cells were infected with *H. pylori* for the times shown. Total cell lysates (**a**) or cytosolic (Cyt) and soluble nuclear (N1) fractions (**b**) were isolated and analyzed for the indicated proteins by IB. A representative blot of at least two experiments was shown.

Supplementary Figure 2: A20 contributes to the down-regulation of alternative NF-κB in *H. pylori* infection
a WT, A20^{KO_1} and A20^{KO_2} AGS cells were infected with *H. pylori* P12 strain. **b** AGS cells were transfected with non-target-specific siRNA (scr) or siRNAs targeting A20 (A20^{si_5} or A20^{si_9}) for 48 h prior to infection with *H. pylori* P1 strain. **c** Same procedure as (b), except NCI-N87 cells were used. (a-c) Total cell lysates were analyzed by IB. A representative blot of at least two experiments was shown.

Supplementary Figure 3: Depletion of TRAF3 has no effect on the interaction of A20 with the TIFA/NIK regulatory complex
a AGS cells were transfected with non-target-specific siRNA (scr) or siRNAs targeting TRAF3 for 48 h prior to infection with *H. pylori*. Total cell lysates were used for IP with an antibody against TIFA or isotype-matched IgG. Eluates were analyzed by IB. A representative blot of at least two experiments was shown.

Supplementary Fig. 1

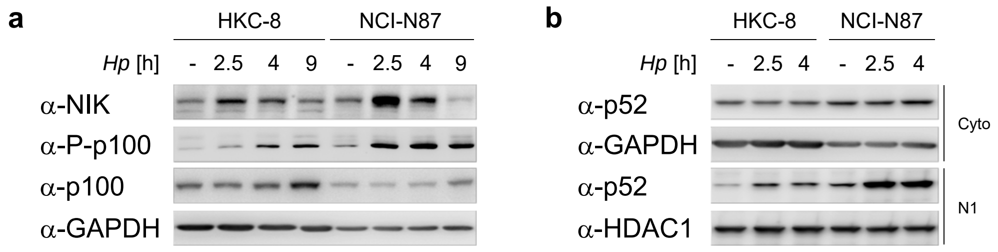

Supplementary Fig. 2

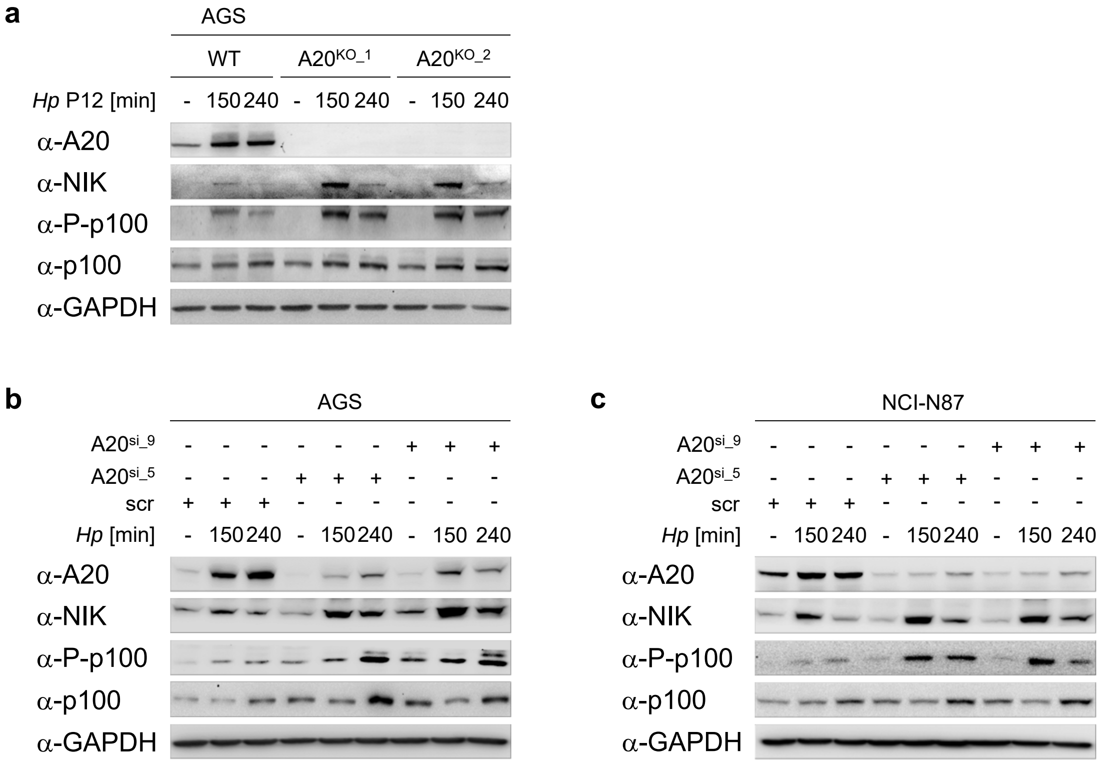

Supplementary Fig. 3

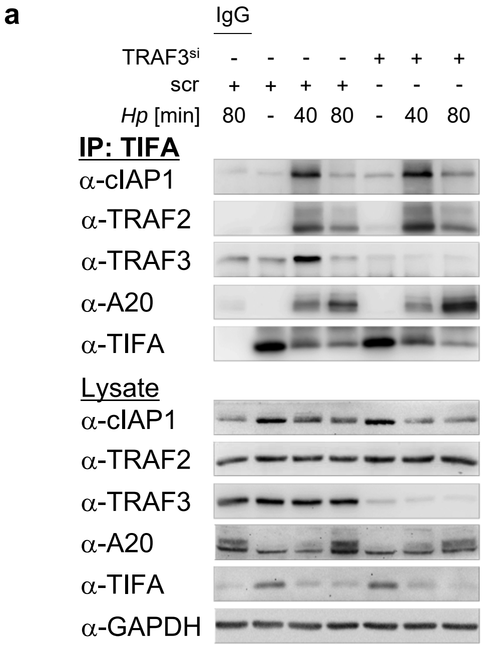

Fig. 1 Uncropped blots

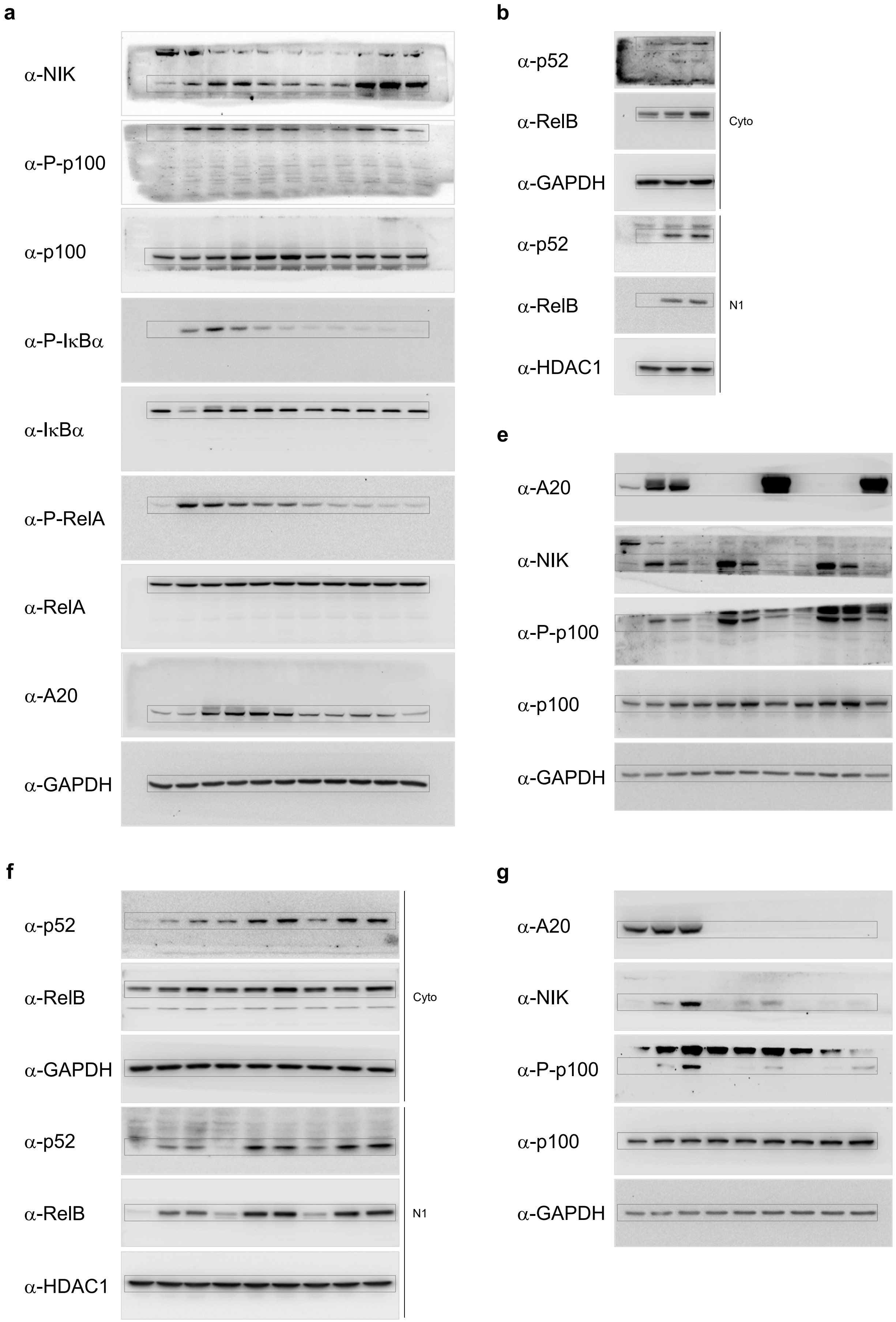

Fig. 2 Uncropped blots

a

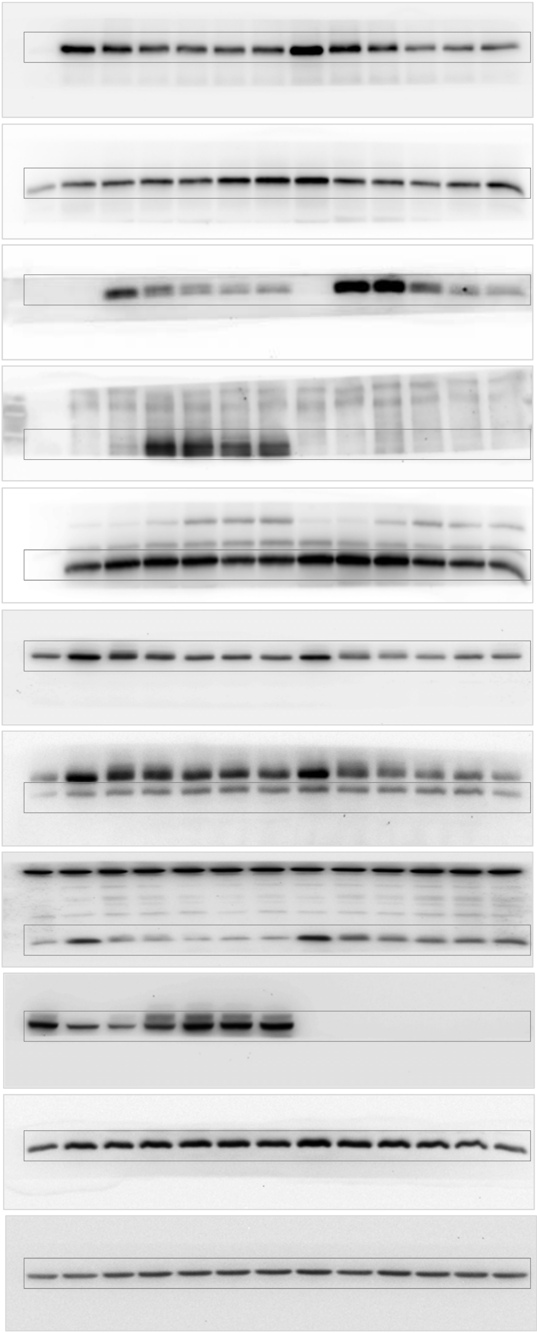

IP: TRAF2
α-cIAP1

α-TRAF3

α-TIFA

α-A20

α-TRAF2

Lysates
*α-cIAP1

*α-TRAF3

α-TIFA

α-A20

α-TRAF2

α-GAPDH

*This is the same IB strip where cIAP1 was probed first, followed by TRAF3

b

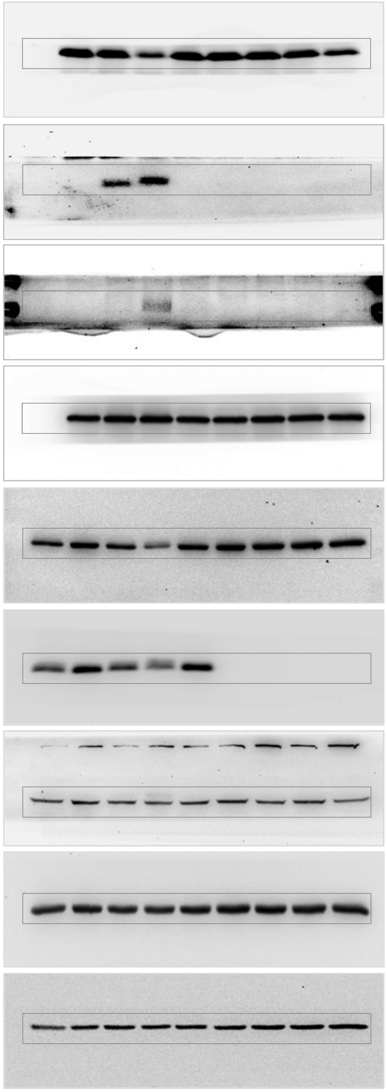

IP: TRAF2
α-cIAP1

α-TIFA

α-A20

α-TRAF2

Lysates
α-cIAP1

α-TIFA

α-A20

α-TRAF2

α-GAPDH

Fig. 2 Uncropped blots

c

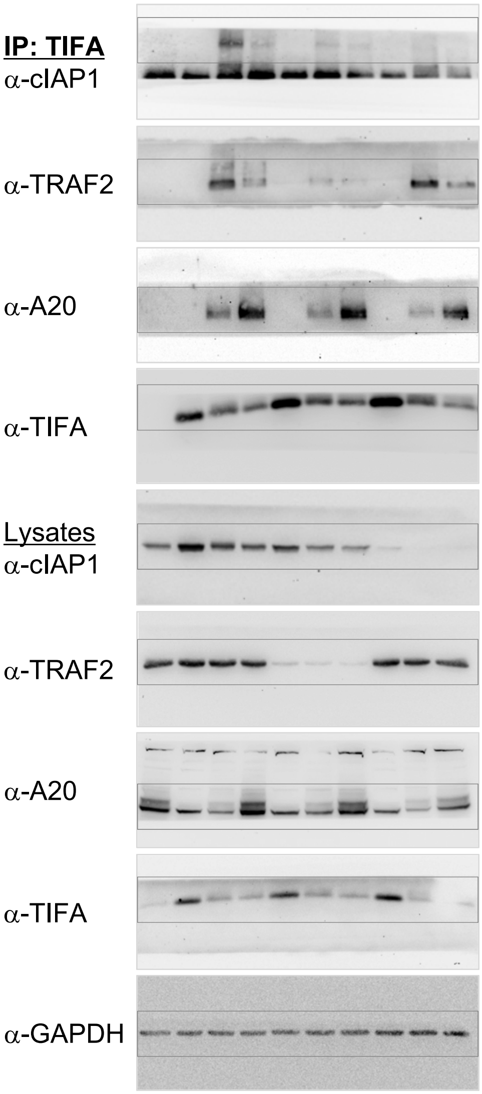

d

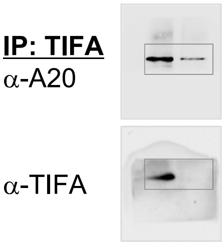

e

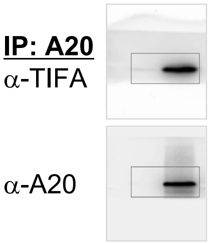

f

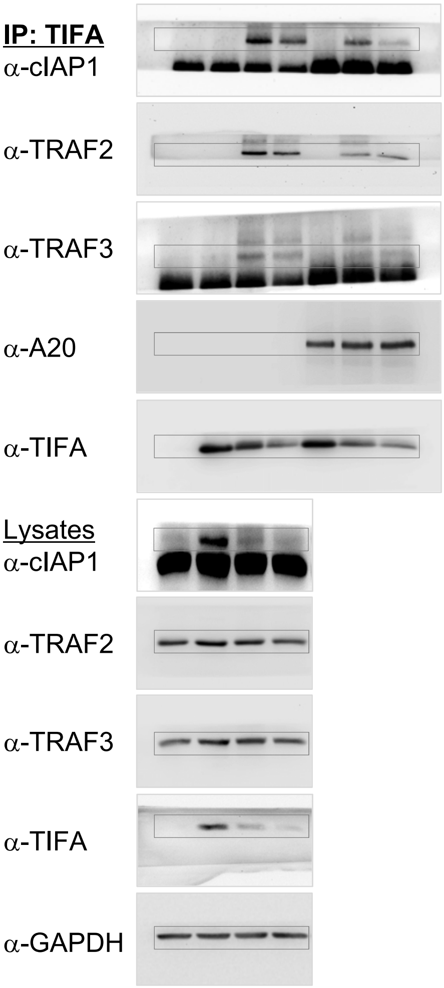

Supplementary Fig. 1

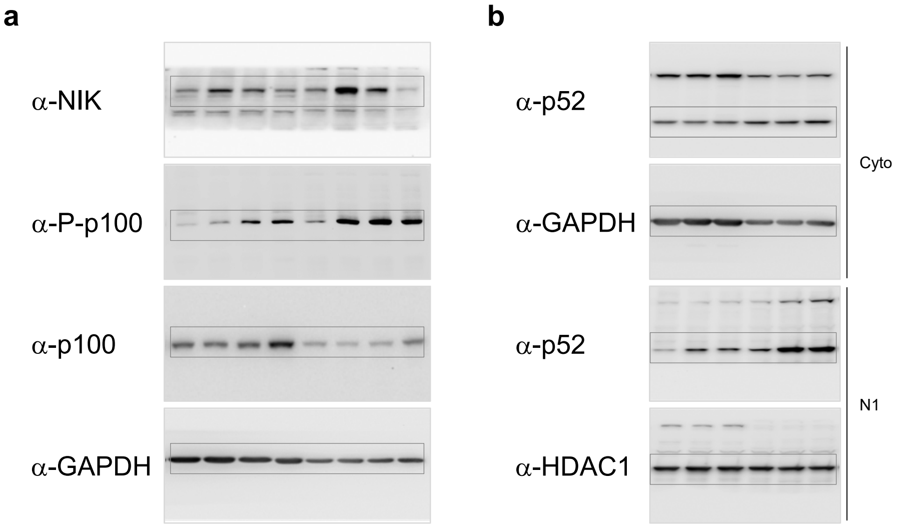

Supplementary Fig. 2

a

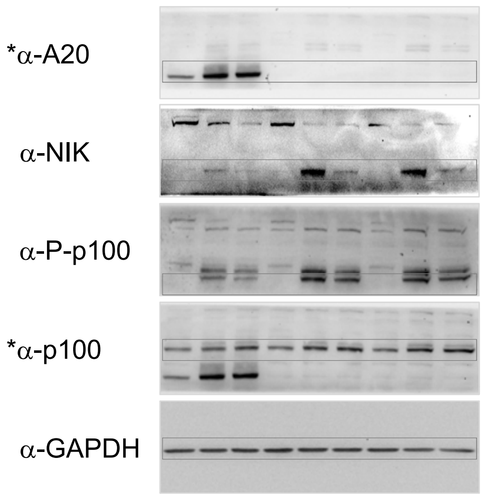

* This is the same IB strip where A20 was probed first, followed by p100

b

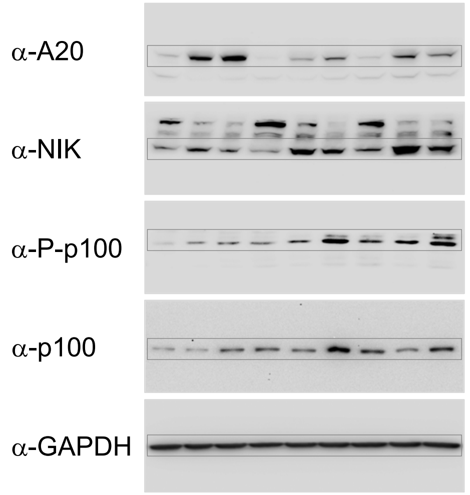

c

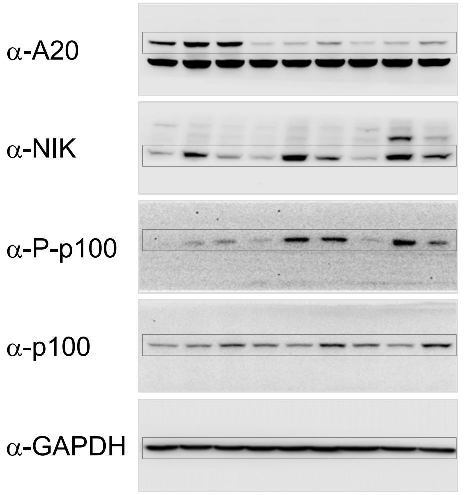

Supplementary Fig. 3

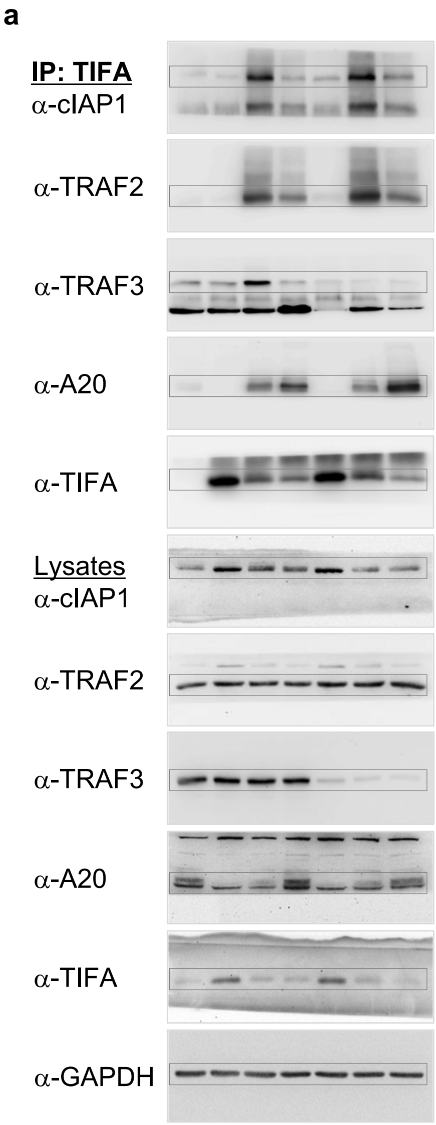

Supplement: Supplementary file 1 — Supplementary file1 (PDF 2769 KB) [file 18_2022_4139_MOESM1_ESM.pdf]
